# Multi-modal ultrasound multistage classification of PTC cervical lymph node metastasis via DualSwinThyroid

**DOI:** 10.3389/fonc.2024.1349388

**Published:** 2024-02-15

**Authors:** Qiong Liu, Yue Li, Yanhong Hao, Wenwen Fan, Jingjing Liu, Ting Li, Liping Liu

**Affiliations:** ^1^ Department of Ultrasound, First Hospital of Shanxi Medical University, Taiyuan, China; ^2^ College of Medical Imaging, Shanxi Medical University, Taiyuan, China; ^3^ Institute of Biomedical Engineering, Chinese Academy of Medical Sciences and Peking Union Medical College, Beijing, China

**Keywords:** papillary thyroid carcinoma (PTC), cervical lymph node metastasis, multi-modal ultrasound imaging, deep learning, DualSwinThyroid model

## Abstract

**Objective:**

This study aims to predict cervical lymph node metastasis in papillary thyroid carcinoma (PTC) patients with high accuracy. To achieve this, we introduce a novel deep learning model, DualSwinThyroid, leveraging multi-modal ultrasound imaging data for prediction.

**Materials and methods:**

We assembled a substantial dataset consisting of 3652 multi-modal ultrasound images from 299 PTC patients in this retrospective study. The newly developed DualSwinThyroid model integrates various ultrasound modalities and clinical data. Following its creation, we rigorously assessed the model’s performance against a separate testing set, comparing it with established machine learning models and previous deep learning approaches.

**Results:**

Demonstrating remarkable precision, DualSwinThyroid achieved an AUC of 0.924 and an 96.3% accuracy on the test set. The model efficiently processed multi-modal data, pinpointing features indicative of lymph node metastasis in thyroid nodule ultrasound images. It offers a three-tier classification that aligns each level with a specific surgical strategy for PTC treatment.

**Conclusion:**

DualSwinThyroid, a deep learning model designed with multi-modal ultrasound radiomics, effectively estimates the degree of cervical lymph node metastasis in PTC patients. In addition, it also provides early, precise identification and facilitation of interventions for high-risk groups, thereby enhancing the strategic selection of surgical approaches in managing PTC patients.

## 1 Introduction

Papillary Thyroid Carcinoma (PTC) is the most common type of thyroid cancer, constituting 85-90% of malignant thyroid tumors. Although PTC progresses slowly with a generally favorable prognosis, the onset of cervical lymph node metastasis in patients can significantly increase the risk of recurrence and distant metastasis, ultimately leading to potential mortality. 2015 American Thyroid Association Management Guidelines for Adult Patients with Thyroid Nodules and Differentiated Thyroid Cancer emphasized that the number of cervical lymph node metastases is a crucial factor in assessing the recurrence risk of thyroid cancer (1). An increase in the number of lymph node metastases corresponds to a poorer clinical outcome for the patient, with a consequent reduction in the 5-year survival rate (2–4).

In the rapidly advancing realm of medical imaging, two principal ultrasonography techniques have emerged as key in predicting cervical lymph node metastasis in thyroid cancer. The first method meticulously examines the primary tumor, while the second method assesses suspicious lymph nodes. The assessment of suspicious lymph nodes to gauge the aggressiveness of thyroid cancer is a direct strategy, but the intricate anatomy of the thyroid gland, coupled with imaging technology limitations, turns preoperative ultrasonography of cervical lymph nodes into a complex task that frequently obstructs the swift identification of suspicious nodes. Therefore, the bulk of research, informed by a pragmatic approach, is derived from studies of the primary tumor, investigating attributes closely linked to the spread of cancer to cervical lymph nodes (5, 6).

The recent technological renaissance has fostered the ascent of radiomics, transcending traditional paradigms of medical imaging analysis. Algorithms meticulously mine imaging data, unveiling hidden information and enabling a comprehensive evaluation of tumor heterogeneity. This forms a foundational framework for the development of precise diagnostic and treatment models, reinforcing the pillars of clinical decision-making. Deep learning stands at the forefront of this innovation, threading significant breakthroughs in computer vision into the fabric of Artificial Intelligence (AI). This technological wonder is extensively applied in medical imaging for tasks such as segmentation, localization, detection, and image fusion, thus elevating the diagnostic precision for pathological changes. Deep learning differs from traditional machine learning—which requires intensive image preprocessing and manual feature identification—by skillfully utilizing raw pixel values from images as input and iteratively refining its models through training (7). However, to our knowledge, there has yet to be any research employing multi-modal ultrasonic radiomics data to develop corresponding deep learning models for evaluating the lymph node status of primary lesions (8, 9).

This study presents DualSwinThyroid, a deep learning classification model meticulously designed for evaluating the invasiveness of thyroid nodules. The model’s ‘Dual’ structure processes multi-modal data, and its ‘Swin’ element utilizes the Swin-Transformer’s (10) advanced image processing capabilities for thorough analysis and extraction of features from ultrasound images. ‘Thyroid’ in its name highlights the model’s specific application to thyroid nodule assessment. DualSwinThyroid is predicated on the Swin-Transformer’s solid framework and is finely calibrated to harness not just its imaging strengths but also to Integrate clinical and ultrasonic data characteristics. Such integration sharpens diagnostic accuracy and enhances efficiency, leading to more targeted and evidence-based treatment plans for patients. Additionally, DualSwinThyroid transcends the conventional binary classification of nodules, introducing a tripartite categorization that corresponds with specific therapeutic approaches and provides a clear framework for complex clinical decision-making processes, aiding in the navigation of diverse treatment alternatives.

## 2 Methods

### 2.1 Patients

**Figure 1:**
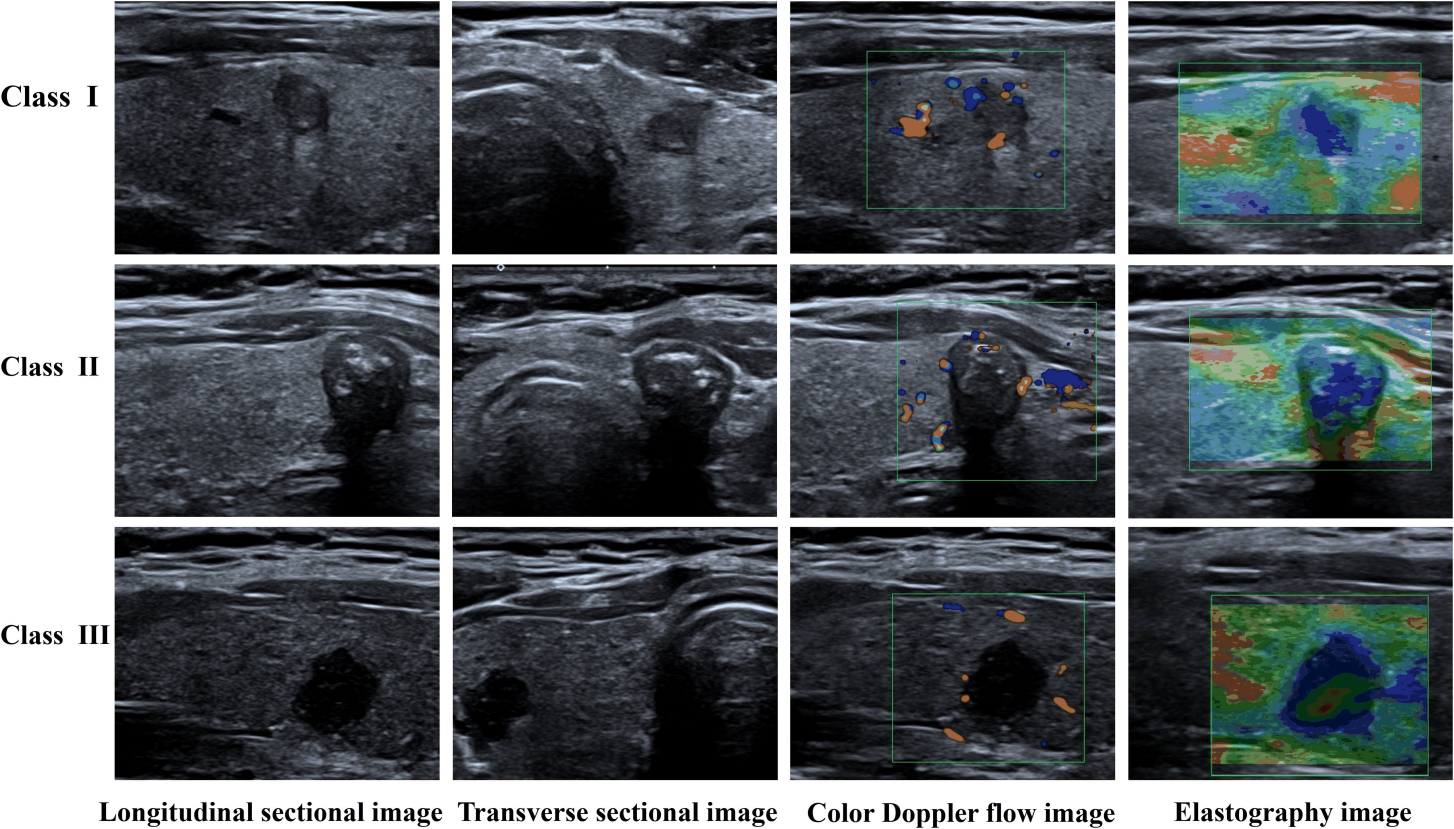
Different categories of image data.

Approval for this retrospective study was obtained from the Ethics Committee of the First Hospital of Shanxi Medical University and the informed consent requirement was waived. Data were gathered from patients who underwent thyroid ultrasonography and subsequent surgical treatment in this hospital from July 2021 to June 2023. Through a rigorous selection process guided by predefined inclusion and exclusion criteria, 299 patients were enrolled, encompassing 339 thyroid nodules captured in 3652 ultrasound images. The postoperative pathological findings served to divide the data into three classes: Class I denoted no lymph node metastasis, Class II included cases with up to five metastatic lymph nodes, and Class III involved cases with more than five metastatic lymph nodes. **Figure 1** displays the types of data images collected.

During data analysis, stringent inclusion and exclusion criteria were applied. The inclusion criteria consisted of: (1) patients who had a total or subtotal thyroidectomy with cervical lymph node dissection; (2) nodules with confirmed surgical pathological diagnoses of papillary thyroid carcinoma; (3) patients who underwent routine ultrasonography and elastography within two weeks before surgery, obtaining clear, complete, and original DICOM images. The exclusion criteria were: (1) patients who received radiofrequency ablation, radiation therapy, or chemotherapy before surgery; (2) ultrasound images of the target tumor marred by artifacts; (3) patients with other malignant tumors; and (4) patients with prior thyroid surgery.

### 2.2 Data collection

All ultrasound scans were performed using the Canon Aplio i800 Color Doppler Ultrasound Diagnostic Device, equipped with an i18LX5 linear wide-band probe with a frequency range of 5-18MHz and real-time ultrasound elastography technology. During routine examinations, patients were positioned supine to expose their necks for scanning. The physician conducted a thorough examination of the thyroid’s bilateral lobes and isthmus, focusing on capturing the echogenicity, dimensions, and vascular flow within the gland. Additionally, for each thyroid nodule, precise records were made of its location, size, composition, echogenicity, shape, margins, presence of hyper-echoic areas, and vascular flow characteristics.

In the ultrasonic elastography examination, the region of interest on the elastographic image was configured to include the entire thyroid lesion and adjacent normal tissue, typically extending 2-3 times beyond the nodule’s size. Patients were asked to hold their breath during the procedure. The physician then positioned the probe perpendicularly on the skin, exerted steady pressure with minimal vibration, and attentively monitored the color patterns displayed on the elastographic images, ensuring to archive the pertinent images.

### 2.3 Data processing

For the purposes of this study, data from 299 patients were included and subsequently randomized into training and testing datasets at a 7:3 ratio. The image data for each patient followed the same categorization protocol. The DualSwinThyroid model underwent training on this dataset and its performance was benchmarked against the Swin Transformer image processing model and the MLP clinical and ultrasound information classification model (11). The construction, training, and prediction of the models were carried out using Python (version 3.8.0). Statistical analysis of the data and calculations of relevant variables were performed using SPSS (version 26.0, IBM Corporation, Armonk, New York).

### 2.4 Statistical analysis

Normality and variance homogeneity tests were conducted for patient characteristics like age and nodule size. Subsequently, the Chi-Squared Test test was utilized to evaluate differences in ultrasonic and clinical features across patient cohorts. Multivariate ordinal logistic regression analysis was applied to determine independent risk factors influencing the extent of PTC lymph node metastasis, with statistical significance established at a two-tailed P-value of less than 0.05. ROC curves were then constructed based on these identified independent risk factors.

### 2.5 Model design and training

**Figure 2:**
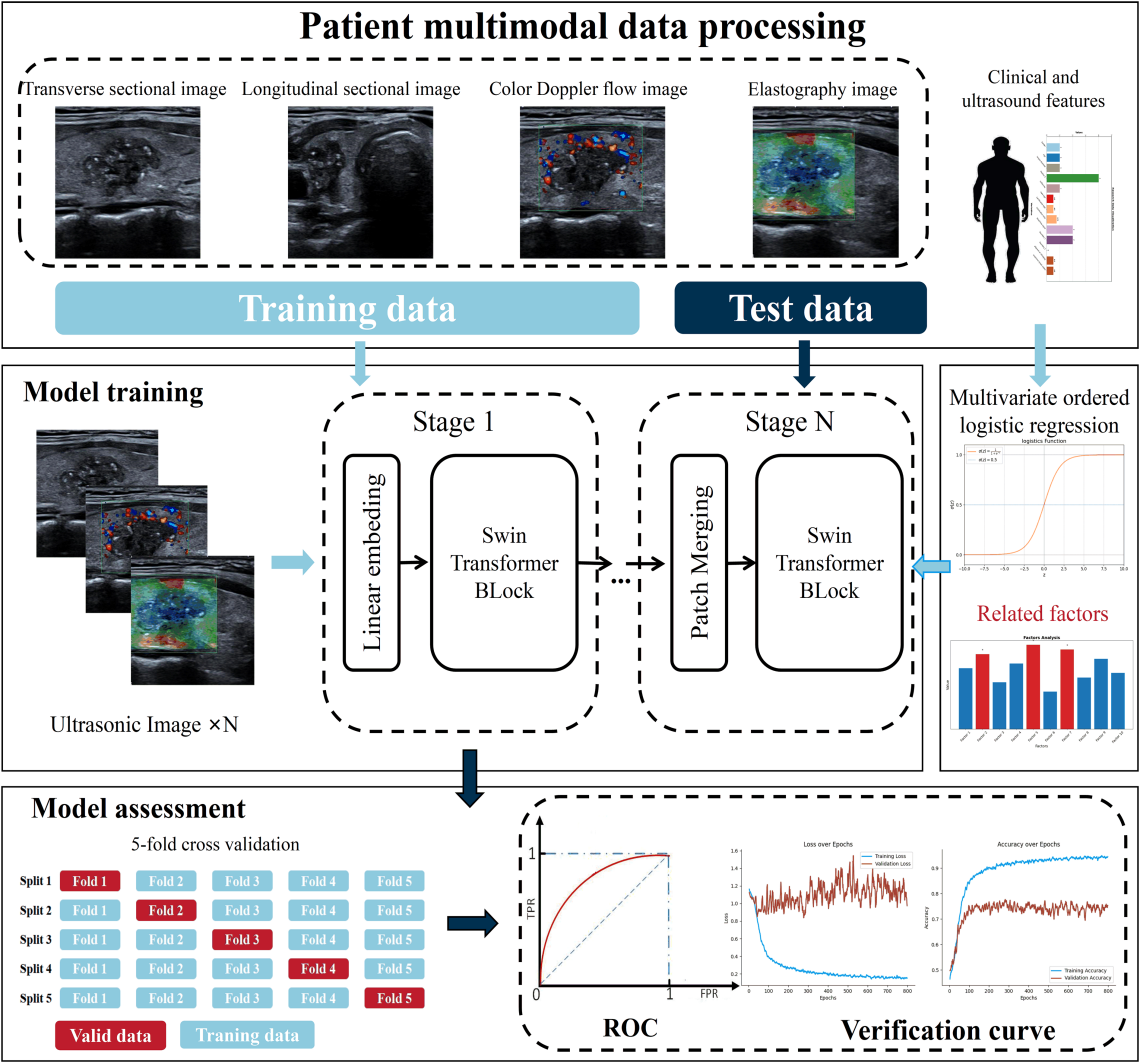
Model training process.

This study utilized data from 299 patients to train the model, with 209 allocated to the training set and 90 to the test set. Additionally, of the 3652 nodule images, 2556 were used for training and 1096 for testing, with the pathological outcomes as the labels for training. It is important to note that the test data were not used during the model’s training phase. The Adam optimizer was employed to train the model across 500 epochs, with a batch size of 16 and an initial learning rate set at 0.0001. The computational work was performed on a platform equipped with an i7-13900F CPU and an RTX 4080TI GPU, and the network architecture was developed on Pytorch 2.0.0+cuda1.18. For more information on the training process, please see **Figure 2**.

#### 2.5.1 DualSwinThyroid model

**Figure 3:**
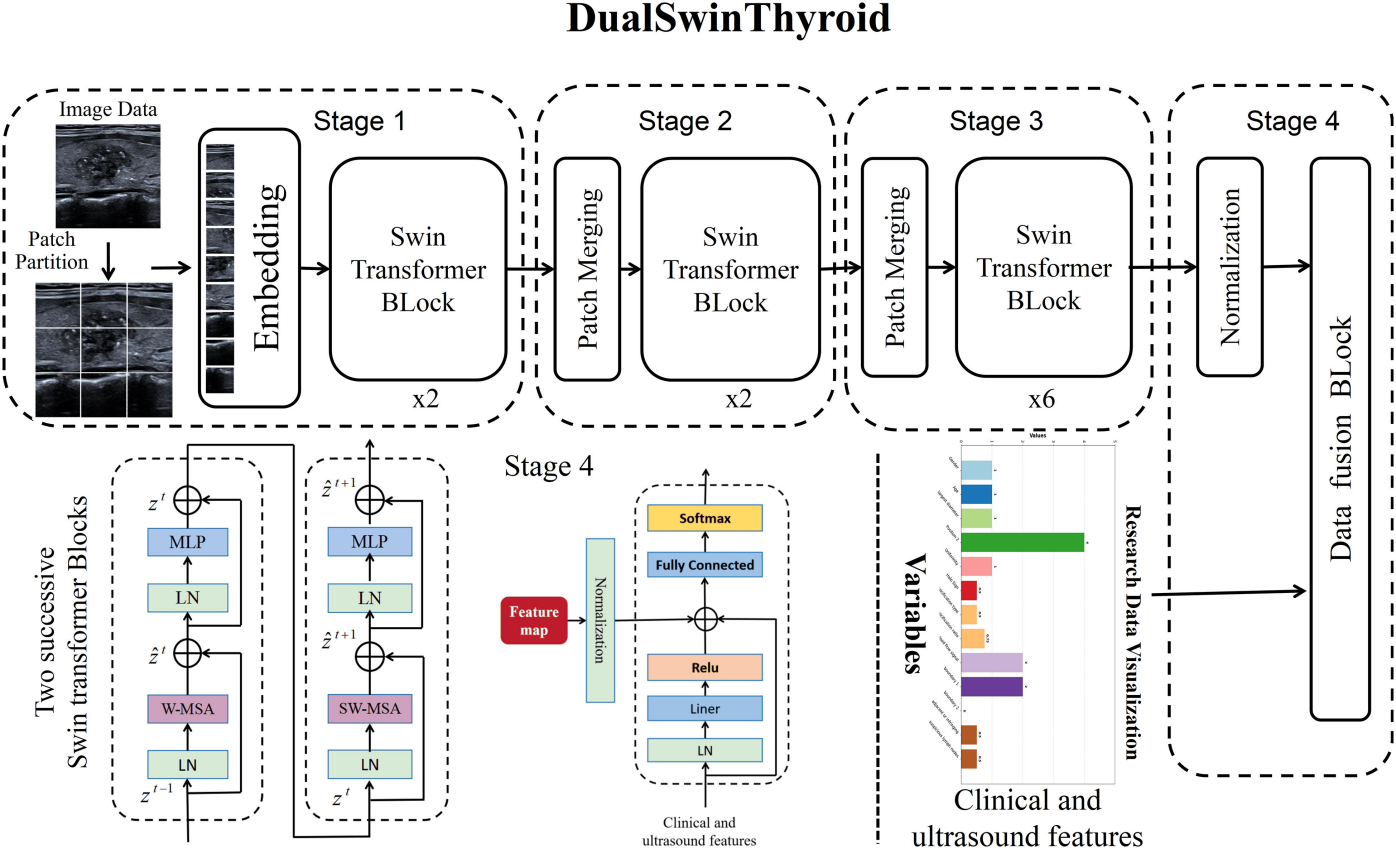
DualSwinThyroid structure diagram.

In this research, the DualSwinThyroid model serves as a deep learning instrument for evaluating thyroid nodule invasiveness and risk levels, detailed in **Figure 3**. It utilizes the Swin-Transformer framework to adeptly process longitudinal sectional and transverse sectional, color Doppler ultrasound and elastographic images. The model operates through three primary image processing stages to extract features deeply and classify invasiveness with precision. Data, once categorized, enters the Data Fusion block, integrating with clinical data vetted through univariate analysis (p-value <0.05). After normalization, this combined data passes through a fully connected layer with a ReLu activation function, proceeds to a subsequent fully connected layer, and culminates in generating predictive probabilities for each category using the Softmax function. Significantly, DualSwinThyroid is capable of processing multiple ultrasound images from the same nodule to produce diagnostic predictions.

#### 2.5.2 Single modality model

A Multi-Layer Perceptron (MLP) model with eight neurons was developed for the classification of clinical and ultrasound data, employing ReLu as the activation function and Softmax for the output layer’s classification purpose. Cross-entropy served as the loss function for optimization. In the training process, the model processed clinical and ultrasound data as inputs, with the features selected based on univariate analyses that produced p-values less than 0.05.

For the classification of images, the Swin-Transformer model was trained, utilizing its Swin-base as the pre-trained model. The input image data were primarily drawn from Regions of Interest (ROI) delineated by physicians during detailed scans and ultrasonic elastography of the thyroid’s bilateral lobes and isthmus.

### 2.6 Evaluation metrics

**Figure 4:**
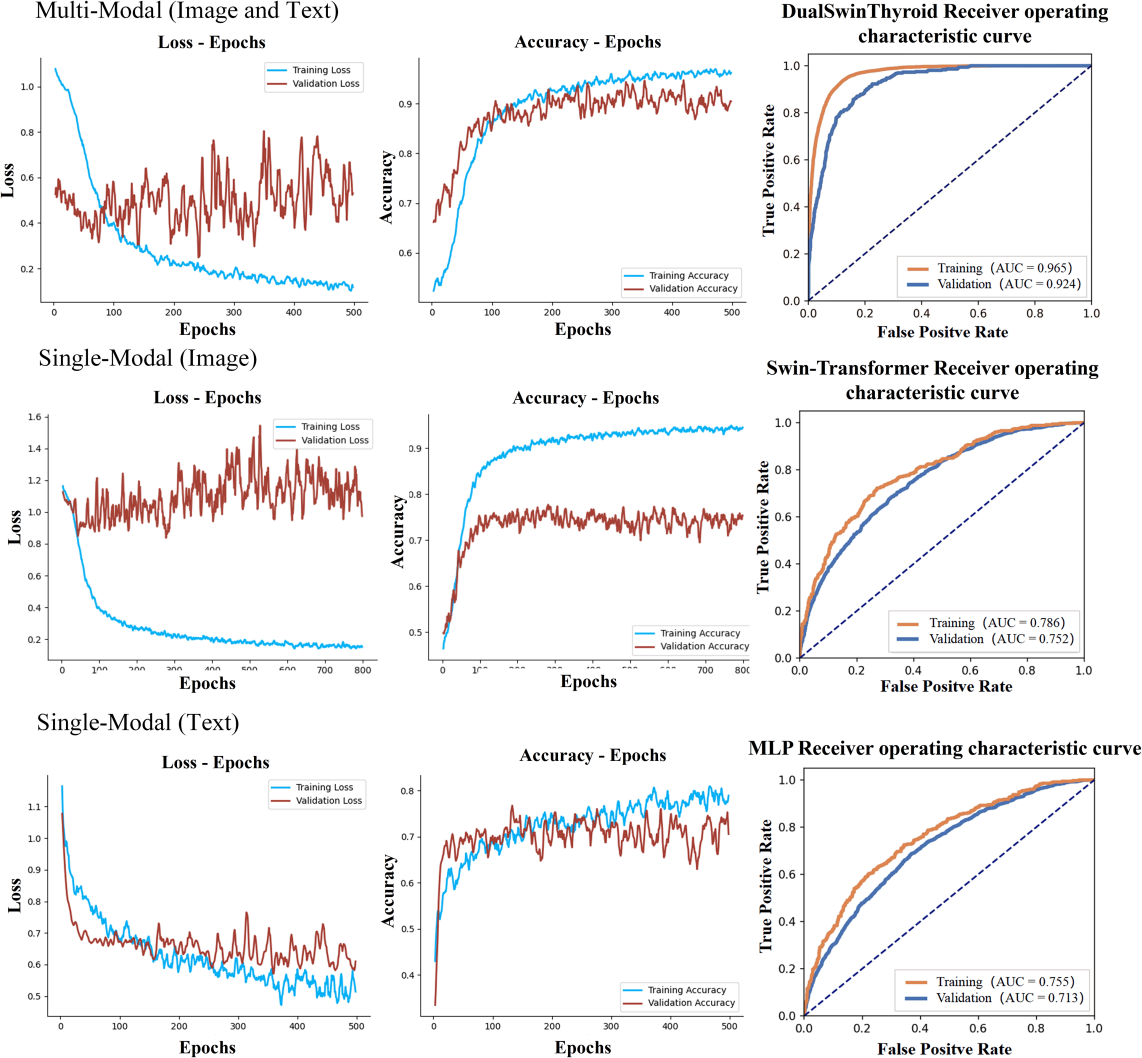
Delineates the training curves of three distinct models. The figure illustrates the training trajectories for multi-modal fusion, single image data, and single text data, each presented with three distinct curves: training loss, training accuracy, and ROC alongside AUC values. The training loss curve gradually stabilizes in the depicted region, contrasting the training accuracy curve, which exhibits significant oscillations. Comprehensively, the ROC curve and AUC values furnish insights into the model's true positive and false positive rates at varying thresholds, notably demonstrating that the multimodal model secures the highest AUC value.

After training each model, Receiver Operating Characteristic (ROC) curves were plotted, and Area Under the Curve (AUC) values were computed for performance evaluation. Algorithms processed ultrasound images of thyroid nodules to accurately determine the extent of metastasis. The test set was then used to further assess the model’s predictive capabilities, including an examination of predicted outcomes and an evaluation of predictive accuracy. Graphs depicting the evolution of prediction accuracy and loss throughout the training were plotted for visual representation, as illustrated in **Figure 4**.

## 3 Results

### 3.1 Data processing results

**Figure 5:**
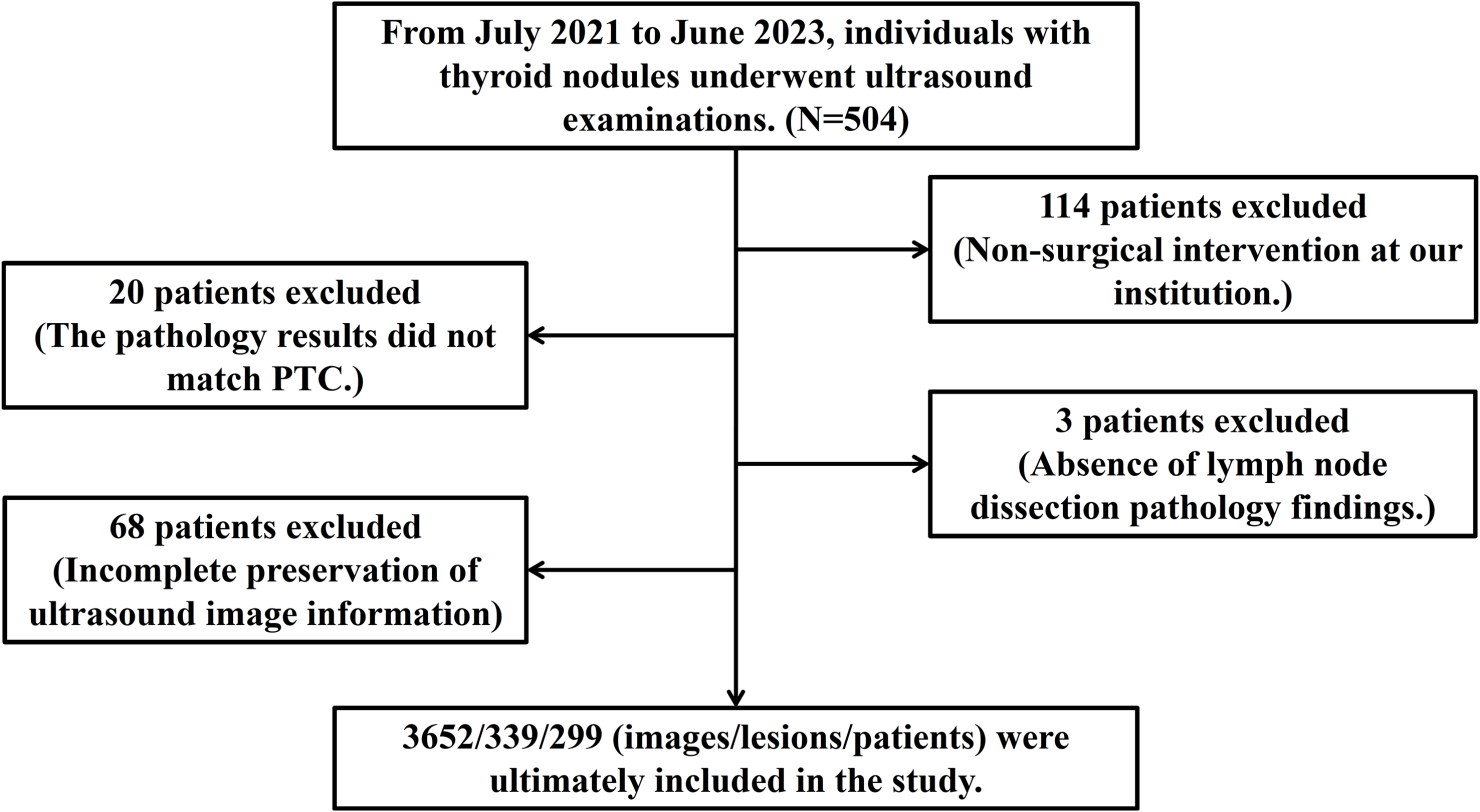
Inclusion and Exclusion diagram.

From July 2021 to June 2023, data from 504 patients who received thyroid ultrasound examinations were initially collected. After rigorous screening, exclusions were applied as follows: 114 cases for the absence of surgery, 20 cases for pathology results not confirming Papillary Thyroid Carcinoma (PTC), 3 cases for missing lymph node dissection records, and 68 cases for incomplete image data. Consequently, the study was narrowed down to 299 cases, which included 339 thyroid nodules and 3652 ultrasound images, along with 19 clinical and ultrasound features, detailed in **Figure 5**.

### 3.2 Statistical analysis results

Univariate analysis assessed clinical and ultrasonic features linked to the degree of cervical lymph node metastasis in Papillary Thyroid Carcinoma (PTC), examining 339 nodules. These were divided into three groups by metastasis count: 158 nodules in Group I, 120 in Group II, and 61 in Group III. The analysis revealed statistically significant differences in various factors, including age, gender, nodule location 2, maximum diameter, boundary, homogeneity, longitudinal-transverse ratio, halo sign, type of calcification, calcification ratio, capsular invasion, blood flow signal, and ultrasound-detectable suspicious lymph nodes. Each factor had P-values below 0.05, which are presented in **Table 1**.

**Table 1:** Univariate analysis of factors associated with cervical lymph node metastasis in PTC.

Variables	Cervical Lymph Node Metastasis Grading			χ^2^	p
	I n (%)	II n (%)	III n (%)		
**Gender**				25.598	<0.001
Female	123(77.85)	90(75.00)	27(44.26)		
Male	35(22.15)	30(25.00)	34(55.74)		
**Age**				14.213	<0.001
>45years	101(63.92)	63(52.50)	22(36.07)		
≤45years	57(36.08)	57(47.50)	39(63.93)		
**Location 1**				6.67	0.154
Right lobe	76(48.10)	63(52.50)	31(50.82)		
Isthmus	3(1.90)	8(6.67)	1(1.64)		
Leftt lob	79(50.00)	49(40.83)	29(47.54)		
**Largest Diameter**				43.635	<0.001
1 cm	124(78.48)	79(65.83)	19(31.15)		
>1 cm	34(21.52)	41(34.17)	42(68.85)		
**Location 2**				17.076	0.009
Upper pole	19(12.03)	16(13.33)	16(26.23)		
Lower pole	32(20.25)	39(32.50)	17(27.87)		
Midsection	101(63.92)	57(47.50)	26(42.62)		
Near isthmus	6(3.80)	8(6.67)	2(3.28)		
**Echo**				7.598	0.107
Hypoechogenic	153(96.84)	116(96.67)	55(90.16)		
very hypoechogenic	0(0.00)	2(1.67)	2(3.28)		
isoechoic	5(3.16)	2(1.67)	4(6.56)		
**Homogeneous**				8.528	0.014
non-uniform	88(55.70)	72(60.00)	47(77.05)		
Uniform	70(44.30)	48(40.00)	14(22.95)		
**Internal Structure**				0.189	0.910
Cystic-solid	3(1.90)	3(2.50)	1(1.64)		
Solid	155(98.10)	117(97.50)	60(98.36)		
**Number of lesions**				3.004	0.223
Unifocal	105(66.46)	70(58.33)	34(55.74)		
Multifocal	53(33.54)	50(41.67)	27(44.26)		
**Boundary**				6.776	0.034
Indistinct	73(46.20)	42(35.00)	33(54.10)		
Distinct	85(53.80)	78(65.00)	28(45.90)		
**Margin**				1.407	0.495
Irregular	79(50.00)	67(55.83)	35(57.38)		
Regular	79(50.00)	53(44.17)	26(42.62)		
**Longitudinal-Transverse Ratio>1**				7.272	0.026
NO	84(53.16)	64(53.33)	44(72.13)		
YES	74(46.84)	56(46.67)	17(27.87)		
**Capsular Invasion**				41.712	<0.001
0	38(24.05)	32(26.67)	3(4.92)		
<0.25	69(43.67)	53(44.17)	14(22.95)		
0.25-0.50	45(28.48)	31(25.83)	34(55.74)		
0.50	6(3.80)	4(3.33)	10(16.39)		
**Suspicious Lymph Nodes**				94.933	<0.001
Yes	16(10.13)	37(30.83)	47(77.05)		
No	142(89.87)	83(69.17)	14(22.95)		
**Halo Sign**				13.639	0.009
Intact	26(16.46)	23(19.17)	2(3.28)		
Partial	68(43.04)	59(49.17)	40(65.57)		
None	64(40.51)	38(31.67)	19(31.15)		
**Calcification Type**				19.064	<0.001
Microcalcification	67(42.41)	59(49.17)	45(73.77)		
Coarse calcification	26(16.46)	13(10.83)	6(9.84)		
No calcification	65(41.14)	48(40.00)	10(16.39)		
**Calcification Ratio**				28.409	<0.001
0	65(41.14)	48(40.00)	9(14.75)		
<0.25	50(31.65)	40(33.33)	17(27.87)		
0.25 - 0.50	27(17.09)	18(15.00)	15(24.59)		
>0.50	16(10.13)	14(11.67)	20(32.79)		
**Elasticity Hardness**				9.648	0.291
Grade I	14(8.86)	11(9.17)	3(4.92)		
Grade II	37(23.42)	26(21.67)	10(16.39)		
Grade III	34(21.52)	28(23.33)	15(24.59)		
Grade IV	68(43.04)	53(44.17)	27(44.26)		
Grade V	5(3.16)	2(1.67)	6(9.84)		
**Blood Flow Signal**				12.144	0.016
Grade I	109(68.99)	71(59.17)	29(47.54)		
Grade II	38(24.05)	41(34.17)	22(36.07)		
Grade III	11(6.96)	8(6.67)	10(16.39)		

Multivariate analysis using ordered logistic regression determined that variables like age over 45 years, a maximum nodule diameter of 1.0 cm or less, male gender, nodule position at the upper and lower poles, specific calcification types, and ultrasound-visible suspicious lymph nodes were statistically significant. Specifically, being over 45 years old, having nodules with a maximum diameter of 1.0 cm or less, microcalcification, coarse calcification, and a longitudinal-transverse ratio of 1 or less were identified as protective factors. In contrast, being male, nodules at the upper and lower poles, and suspicious lymph nodes on ultrasound were established as independent risk factors. These findings are elaborated in **Table 2**.

**Table 2:** Ordered regression analysis of features associated with the extent of cervical lymph node metastasis in PTC.

Variables	B	S.E	Wald	P
I	-14.539	0.795	334.852	<0.001
II	-12.08	0.816	219.372	<0.001
Age (>45years)	-0.577	0.236	5.979	0.014
Gender (Male)	0.837	0.254	10.840	<0.001
Largest Diameter (≤1.0cm)	-0.791	0.318	6.19	0.013
Boundary (Indistinct)	0.206	0.253	0.661	0.416
Longitudinal-Transverse Ratio >1 (NO)	-0.134	0.261	0.262	0.608
Homogeneous (Uniform)	0.128	0.286	0.199	0.655
Location 2				
Near isthmus	-0.152	0.56	0.074	0.786
Upper pole	0.927	0.339	7.480	0.006
Lower pole	0.826	0.274	9.110	0.003
Midsection	–	–	–	–
Suspicious Lymph Nodes (YES)	2.164	0.29	55.567	<0.001
Halo Sign				
Intact	-0.219	0.386	0.32	0.572
Partial	0.079	0.282	0.079	0.779
None	–	–	–	–
Calcification Type				
Micro-Calcification	-15.216	0.495	943.168	<0.001
Coarse-Calcification	-14.108	0.448	993.29	<0.001
No-Calcification	–	–	–	–
Calcification Ratio				
0	-14.99	0	–	–
<0.25	-0.781	0.419	3.469	0.063
0.25-0.50	-0.627	0.42	2.234	0.135
>0.50	–	–	–	–
Capsular Invasion				
0	0.099	0.613	0.026	0.872
<0.25	0.054	0.571	0.009	0.924
0.25-0.50	0.031	0.547	0.003	0.955
>0.50	–	–	–	–
Blood Flow Signal				
Grade I	0.402	0.455	0.781	0.377
Grade II	0.613	0.464	1.746	0.186
Grade III	–	–	–	–

### 3.3 Model performance results

The development of the DualSwinThyroid model incorporated a 5-fold cross-validation approach to optimize hyperparameters. The model’s predictive performance was thoroughly evaluated, tracking not just the loss curve but also deriving accuracy metrics from the test set. The ROC curve was plotted, and the AUC value was calculated, as shown in **Figure 4** (Training Curve). Additionally, essential metrics like sensitivity and specificity were analyzed.

**Figure 6:**
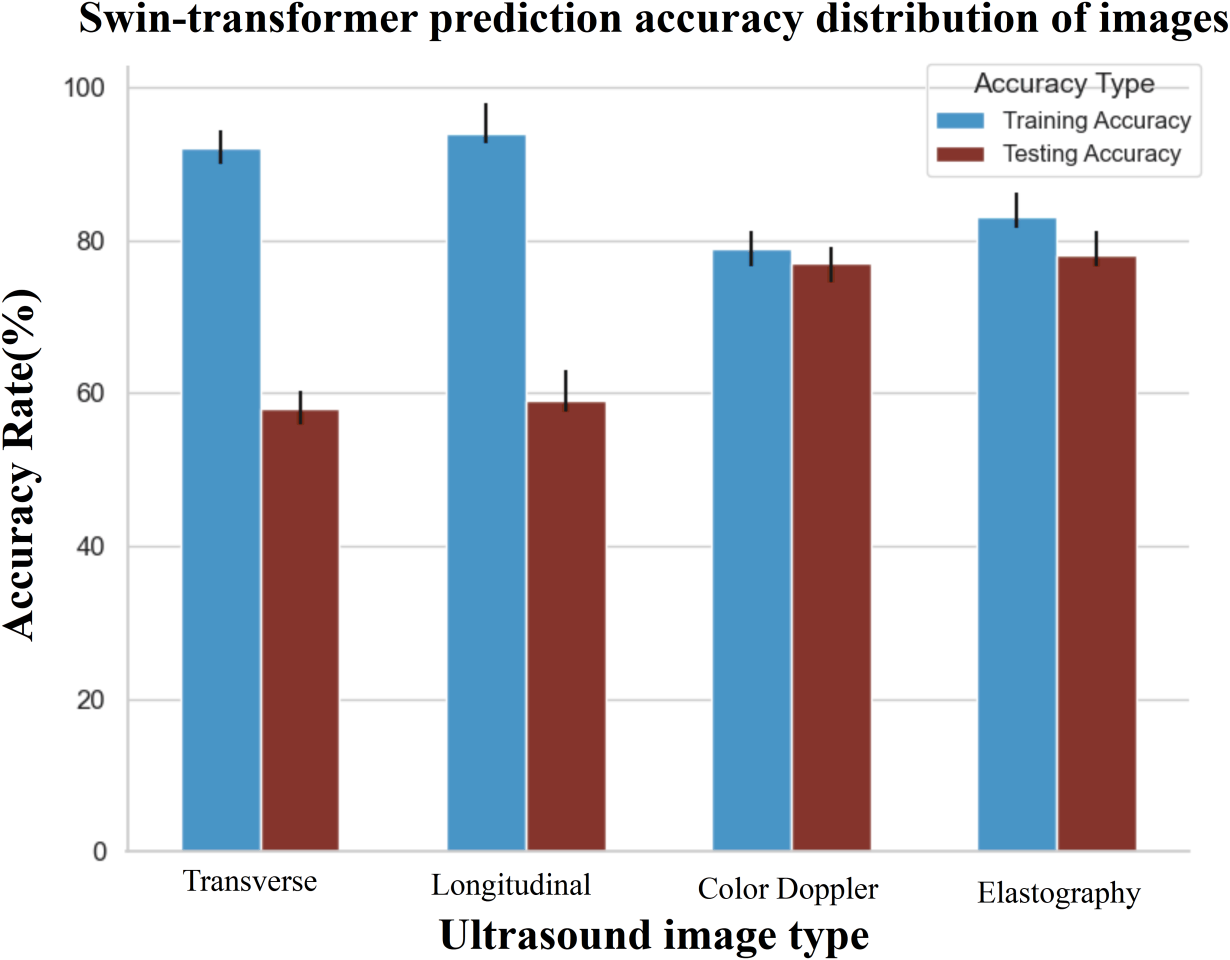
The prediction accuracy of different data in the corresponding model. The evaluation indicates that multi-modal data performs optimally within the test set. Specifically, when considering single modality data, the accuracy of Doppler ultrasound images and elastography images within the test set surpasses that of transverse and longitudinal images.

In training the single-modality models, training accuracy and loss were also monitored, as shown in **Figure 4**. A significant observation was the Swin-Transformer’s classification results varying considerably with different image data types in the test set. Color Doppler ultrasound and elastography images yielded higher classification accuracy than transverse and longitudinal images, as evidenced by superior accuracy in **Figure 6** (Classification Performance).

## 4 Discussion

In Papillary Thyroid Carcinoma (PTC), cervical lymph node metastasis is a common occurrence, with an estimated 40-90% of PTC patients potentially experiencing such metastasis (12, 13). In 2015, the American Thyroid Association issued management guidelines for adult thyroid nodules and differentiated thyroid cancer patients, highlighting the degree of cervical lymph node metastasis as a significant indicator of thyroid cancer recurrence. To curb the rapid progression of PTC, a crucial step is early identification of tumors with metastatic potential in clinically diagnosed PTC patients. Surgical removal is regarded as the primary treatment modality (14). For patients at risk of suspected lymph node metastasis, prophylactic central or lateral neck dissection is recommended. When performing prophylactic lymph node dissection and total thyroidectomy, the increased risk of postoperative complications, especially hypoparathyroidism, must be considered. The study by Henry et al. (15) reported that central neck lymph node dissection could escalate the risk of permanent hypoparathyroidism from 0% to 4%. However, research by Nixon I J et al. indicates that reoperation post PTC recurrence is relatively challenging, significantly elevating surgical complications and impacting the quality of life of patients (16). Hence, early identification of cervical lymph node metastasis in PTC not only aids in clinically selecting the appropriate surgical plan and scope, reducing the occurrence of postoperative complications, but also in minimizing recurrence risks, averting secondary surgeries, and proactively improving prognosis. Nonetheless, the sensitivity of solely relying on ultrasonographic characteristics to indicate lymph node metastasis remains insufficient, with some lymph node metastases exhibiting unremarkable ultrasonic features - a common scenario in clinical practice. Studies also revealed a mere 33% sensitivity of ultrasound in detecting central lymph node metastasis (17). Preoperative neck ultrasonography is inevitably influenced by inter-observer variability, thereby rendering the diagnostic outcome lacking in certain objectivity.

This study conducted a thorough investigation into the independent risk factors for cervical lymph node metastasis in Papillary Thyroid Carcinoma (PTC), analyzing 3,652 multi-modal ultrasonographic images and data from 299 patients. The results underscore the need for increased attention to cervical lymph node metastasis and recurrence risk, particularly in male patients, those aged 45 or younger, with thyroid cancer nodules larger than 1cm, nodules at the lower pole and center of the gland, presence of calcification, and suspicious lymph nodes detected by ultrasonography. To improve the accuracy of predicting cervical lymph node metastasis in PTC, the study introduced the DualSwinThyroid Model, a deep learning tool combining ultrasonographic images with clinical data. The model achieved a high AUC of 0.924, with an accuracy rate of 96.3%, and commendable sensitivity and specificity, highlighting its potential as an effective non-invasive assessment tool for lymph node involvement in PTC. Model performance is detailed in **Table 3**.

**Table 3:** Performance comparison of models in this study.

Model	AUC	Acc (%)	Sen (%)	Precision	F1 Score
Single-Modal (Image)	0.713	75.74	77.48	58.59	65.81
Single-Modal (Text)	0.752	77.53	78.77	60.85	67.80
**DualSwinThyroid (Image and Text)**	**0.924**	**96.35**	**99.45**	**97.93**	**98.66**

*Bold font denotes the predictive performance of the optimal model in this study.

Earlier research has typically hinged on machine learning and statistical methods, analyzing image, clinical, and ultrasonic features in isolation, without an in-depth approach (18–23). Luchen Chang et al. employed deep learning, alongside ultrasonic and clinical data, to develop a composite nomogram for predicting central lymph node metastasis in PTC patients, using grayscale images for radiomics and six related features. However, the absence of multi-modal ultrasonic data and additional correlating factors slightly impeded the predictive accuracy (22). Fu Li et al. achieved promising results in forecasting cervical lymph node metastasis using conventional machine learning models (19). These models, however, sometimes fail to discern complex patterns in large datasets, particularly with fluctuating data distributions or noise, which can hinder their generalization. Additionally, they depend on manual feature processing, which can compromise performance if not done meticulously. Notably, most current studies focus on merely detecting cervical lymph node metastasis, which affects the precision of choosing surgical methods and predicting patient outcomes. Details on comparative studies can be found in **Table 4**.

**Table 4:** Comparison among the prediction models of PICC-RT.

Research Group	Predicted Performance	Research data	Research objectives	Number of risk factors	Prediction Method
Jinhua Yu et al. (18)	AUC=0.90-0.93	3172(I)	Prediction of LNM in PTC (2 classification)	--	TLR
Fu Li et al. (19)	AUC=0.803 Sensitivity = 0.727 Specificity=0.800	126(P)	Prediction of LNM in thyroid cancer (2 classification)	1079 (Radiomics Features)	Machine Learning
Jia Zhan et al. (20)	AUC=0.757	405(P)	Prediction of LNM in PTC (2 classification)	3	logistic regression
Wen-Hui Li et al. (21)	AUC=0.838	450(P)	Prediction of cervical LNM in patients with mPTMC (2 classification)	6	Nomogram
Luchen Chang et al. (22)	AUC=0.809	3359(I)	Predicting CLNM. (2 classification)	6	Nomogram
Chenxi Liu et al. (23)	AUC=0.669	966(P)	Prediction of LNM in PTC (2 classification)	5	logistic regression
**Ours**	**AUC=0.924** **ACC = 96.35%**	**3652(I)/299(P)**	**Prediction of LNM in PTC** **(3 classification)**	**13**	**Deep Leaning**

*Bold font denotes the predictive performance of the model in this study.

P, Patients data; I, Image data.

The DualSwinThyroid model introduces a refined thyroid nodule management method by categorizing them into three different levels to enhance clinical decision-making accuracy. For Class I nodules without lymph node metastasis, unilateral lobectomy and isthmectomy are advised as the primary surgical path. In Class II cases with less than five metastatic lymph nodes, the model recommends total or near-total thyroidectomy and ‘selective’ cervical lymph node dissection, in conjunction with clinical judgment. The term ‘selective’ cervical lymph node dissection, as used here, refers to a process where the surgeon, integrating systematic preoperative evaluation with intraoperative biopsy pathology, determines the necessity of lymph node dissection and identifies the specific regions for dissection, thereby minimizing the risk of secondary surgical interventions. For Class III nodules with more than five metastatic lymph nodes, total or near-total thyroidectomy and extensive cervical lymph node dissection are advised, which is more extensive than standard elective central neck dissection, especially in cases of widespread metastasis. Physicians expand the scope of dissection based on a comprehensive assessment of other clinical indicators. This recommendation balances comprehensive treatment and the risk of overtreatment. The DualSwinThyroid model’s multi-tiered classification not only demonstrates diagnostic accuracy but also conforms to contemporary medical guidelines, enhancing patient-centered surgical decision-making.

Throughout the development of our model, several key findings emerged. Initially, it was apparent that the accuracy of predictions using single-modality data alone was relatively low, with image data outperforming clinical and ultrasonic data, highlighting the importance of imaging in predictive models. In the training dataset, accuracy confidence was higher for transverse and longitudinal images than for color Doppler and elastography. Yet, this pattern shifted in the testing phase, where color Doppler and elastography images achieved greater accuracy, revealing variable performance across imaging types during different phases. Moreover, integrating four types of images—transverse, longitudinal, color Doppler, and elastography—with clinical and ultrasonic data provided the most accurate predictions for individual cases. Adding more images did not improve but rather slightly reduced the predictive performance. These findings have been instrumental in fine-tuning the model and determining the most effective imaging techniques to increase accuracy, setting a course for future advancements in model enhancement.

This study successfully developed a multi-modal ultrasound radiomics deep learning model for predicting cervical lymph node metastasis in PTC. It aims to leverage machine learning to identify features related to metastasis, thereby improving surgical decision-making in PTC. However, the work is retrospective and exploratory in nature. Moreover, its scope is limited by its single-institution design, which might not fully represent a wider patient population. Currently, the final pathological assessment of the extent of lymph node metastasis still relies on the thoroughness and accuracy of surgical removal. While the prediction model offers a potential foundation for clinical decision support, its benefits are yet to be confirmed in prospective clinical trials. Therefore, the anticipated advantages, such as reducing lymph node dissections, financial burden, and supporting emerging practitioners, are promising but need further validation in various clinical settings. Future efforts will focus on expanding data sources and rigorously validating the model’s clinical utility in different institutions.

## Data availability statement

The raw data supporting the conclusions of this article will be made available by the authors, without undue reservation.

## Ethics statement

The studies involving humans were approved by First Hospital of Shanxi Medical University. The studies were conducted in accordance with the local legislation and institutional requirements. The ethics committee/institutional review board waived the requirement of written informed consent for participation from the participants or the participants’ legal guardians/next of kin because retrospective nature of the study: if the study is retrospective, analyzing previously collected data, written informed consent may not be required as the data is often de-identified and does not pose a risk to the privacy of the individuals involved.

## Author contributions

QL: Funding acquisition, Investigation, Methodology, Writing – original draft, Writing – review & editing. YL: Data curation, Methodology, Software, Visualization, Writing – original draft. YH: Investigation, Supervision, Writing – original draft. WF: Investigation, Supervision, Validation, Writing – review & editing. JL: Supervision, Validation, Writing – original draft. LL: Conceptualization, Formal analysis, Funding acquisition, Supervision, Writing – original draft, Writing – review & editing. TL: Formal analysis, Supervision, Validation, Writing – original draft, Writing – review & editing.
